# Identifier Mapping in Cytoscape

**DOI:** 10.12688/f1000research.14807.2

**Published:** 2018-08-06

**Authors:** Adam Treister, Alexander R. Pico

**Affiliations:** 1 Institute of Data Science and Biotechnology, Gladstone Institutes, San Francisco, CA, 94158, USA

**Keywords:** Cytoscape, ID Mapping, Identifiers, BridgeDb

## Abstract

Identifier Mapping, the association of terms across disparate taxonomies and databases, is a common hurdle in bioinformatics workflows. The *idmapper* app for Cytoscape simplifies identifier mapping for genes and proteins in the context of common biological networks. This app provides a unified interface to different identifier resources accessible through a right-click on the table's column header. It also provides an OSGi programming interface via *Cytoscape Commands* and *CyREST* that can be utilized for identifier mapping in scripts and other Cytoscape apps, and supports integrated Swagger documentation.

## Introduction

**Figure 1.**
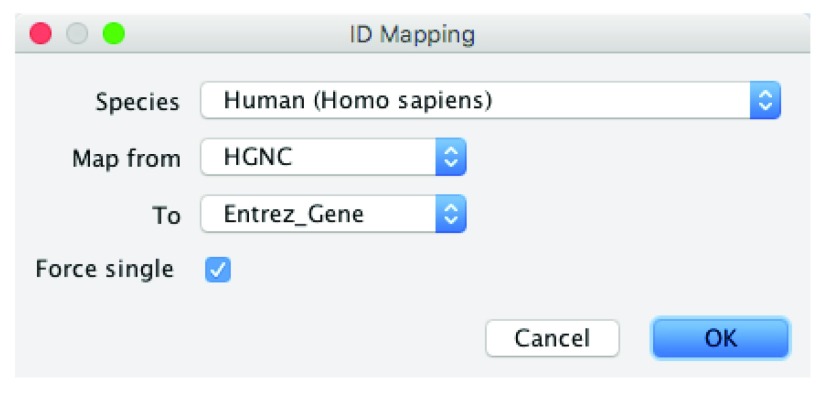
Simplified dialog for ID Mapping. Four options are presented to the user when accessing idmapper from within the Cytoscape GUI, each with common default or inferred values to reduce the number of steps required of the user.

Cytoscape is an integrated network visualization tool and analysis platform ^1, 2^. Within its common workflows, identifier mapping remains a challenge when working with biological data from different sources. This problem has been addressed by the BridgeDB project ^3^, which created clients and services to translate between various identifiers. The original BridgeDb app ^4^ for Cytoscape was written to provide an exhaustive set of functions to match the full capabilities of BridgeDb. Though this provided the needed functionality, its basic usage was unnecessarily complex. The idmapper app is a useful alternative, providing access to a commonly used subset of BridgedDb databases via web services by means of a simplified interface bundled into Cytoscape. Now, without any installation or configuration, Cytoscape users can right-click on a table header to map that column’s data to a different namespace ( Figure 1). Although, the breadth of coverage is smaller than the full-featured BridgeDb app, it still covers over a dozen identifier data sources maintained by BridgeDb, including Ensembl, Entrez Gene, HGNC, KEGG, Uniprot-TrEMBL and various species-specific sources. Because idmapper supports Cytoscape’s new CyREST interface, identifier mapping can be included in scripted workflows, and driven from R or python programs.

## Implementation

### Inferring the data source

From within Cytoscape, a user initiates an ID mapping operation by right-clicking on the header of a column containing identifiers in the *Table Panel*. Based on the specified species a list of data sources is provided to the user. In the most common cases the type of identifier can be guessed by idmapper based on the its format and is presented as the default selection. Table 1 shows the supported data sources and example identifier formats. The app looks at the first ten entries and chooses the source that matches corresponding regular expressions provided by BridgeDb. If there is no match (or if more than one system is matched), then it simply chooses first option in the list as the default selection.

**Table 1.** Supported Data Sources. The parameter names of supported data sources, their species exclusivity and an example identifier. Note that Ensembl support is only for gene identifiers, not proteins.

Data Source	Species	Example
Ensembl	*Any*	ENSG00000139618
Entrez Gene	*Any*	11234
KEGG Genes	*Any*	syn:ssr3451
UniGene	*Any*	Hs.553708
Uniprot-TrEMBL	*Any*	P62158
FlyBase	Drosophila melanogaster	FBgn0011293
HGNC	Homo sapiens	DAPK1
MGI	Mus musculus	MGI:2442292
RGD	Rattus norvegicus	2018660
SGD	Saccharomyces cerevisiae	S000028457
TAIR	Arabidopsis thaliana	AT1G01030
WormBase	Caenorhabditis elegans	WBGene00000001
ZFIN	Danio rerio	ZDB-GENE-041118-11

### Cytoscape tasks

There are two different tasks supported by the idmapper app. *ColumnMappingTask* is activated by the right-click mouse event on a table header. It infers the current table and column from the information that comes from the mouse event, triggering a dialog (see GUI use case) that collects the information needed to make a call to BridgeDb web services. Please refer to the BridgeDb project for details about their services and sources ^3^. In order to support automation, we added *MapColumnCommandTask* as an analog that is exposed specifically for *Commands* and *CyREST* access. These tasks eventually result in the same algorithms being invoked.

## Use cases

### Cytoscape graphical user interface (GUI)

The idmapper app provides the same basic functionality of the BridgeDb app with less fuss. Users do not have to install it, launch it, make configuration decisions or think about which database they are accessing. The app comes bundled with every Cytoscape release. As such its usage in Cytoscape via the interactive GUI (graphical user interface) is documented in the Cytoscape manual: http://manual.cytoscape.org/en/stable/Node_and_Edge_Column_Data.html#mapping-identifiers.

To map an identifier from one source to another, right click on the column header of your identifier. Select the option to **Map Column** to bring up the idmapper dialog ( Figure 1).

The idmapper dialog presents a few choices the user can override before performing ID mapping. The default ***Species*** is determined by the previous selection made by the user per network, providing a persistant behavior across mulitple searches. The available choices for the identifier data sources are determined by the species. The ***Map from*** data source is automatically selected based on an inspection of the first ten identifiers found in the column clicked on by the user. This can be overridden by the pull down menu. The ***To*** data source must be selected by the user; Ensembl is presented by default. Finally, the ***Force single*** checkbox offers to simplify the results of ID mapping by ignoring one-to-many cases and only keeping the first result (arbitrarily determined by the BridgeDb web service result). If the option is off, a list of results will appear in the column. This can easily be overridden by clicking the toggled checkbox. The result of the mapping is appended to the node table in a column named after the target data source, e.g., "Ensembl". If a column by that name already exists, a parenthesized number is appended to the name to ensure it is unique, e.g., "Ensembl(1)".

### Cytoscape command line interface

The command interface does not use the same tasks as the GUI. In the GUI use case, the app knows the current context of where the command was activated, i.e., the network, table and column. This information must explicitly be provided as paramaters to the command interface to perform the same operation. Thus, in addition to species, mapFrom, mapTo and forceSingle, the command line operation of idmapper also requires networkName, table and columnName (see next section for more details).

### Cytoscape automation

**Figure 2.**
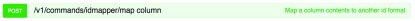
Swagger documented function. The functionality of idmapper is contained in this single function: *map column*.

In the scripting environment, idmapper provides all of its functionality in a single call ( Figure 2). This means that identifier mapping can be incorporated into Cytoscape automation workflows with a single additional command. The scripting version of the command includes extra parameter for **columnName**, **networkName** and **table**, which are implicit in the GUI version from the location of the mouse event.

The **map column** function takes the following parameters:

- ***columnName*** (string): Specifies the column name where the source identifiers are located
- ***forceSingle*** (string, optional): When multiple identifiers can be mapped from a single term, this forces a singular result
- ***mapFrom*** (string): Specifies the data source describing the existing identifiers
- ***mapTo*** (string): Specifies the data source identifiers to be returned as a result in a new column
- ***networkName*** (string, optional): Which network is used in the mapping.
- ***species*** (string): The common *or latin* name of the species to which the identifiers apply, e.g., Human, *Homo sapiens*, Mouse, *Mus musculus*, Rat, *Rattus norvegicus*, Frog, *Xenopus tropicalis*, Zebra fish, *Danio rerio*, Fruit fly, *Drosophila melanogaster*, Mosquito, *Anopheles gambiae*, Arabidopsis, *Arabidopsis thaliana*, Yeast, *Saccharomyces cerevisiae*, E. coli, *Escherichia coli*, Tuberculosis, *Mycobacterium tuberculosis*, Worm, *Caenorhabditis elegans*
- ***table*** (string, optional): Which table is used as the source of the identifiers, e.g., "node" for the default node table

With Cytoscape running, the **map column** function can be called from any scripting environment or programming language that supports REST calls. In the case of R and Python scripts, there are dedicated packages to make this even easier. The *RCy3* package wraps this command in an R function called **mapTableColumn** to conform to other table functions ( https://www.bioconductor.org/packages/release/bioc/html/RCy3.html). The *py2cytoscape* library similarly provides this command as a python function, **cyclient.idmapper.map_column** ( https://github.com/cytoscape/py2cytoscape). The advantage of using one of these dedicated packages is having more concise syntax and language-specific conventions. In RCy3, for example, the custom **mapTableColumn** function simplifies the call, conforms to other RCy3 functions and returns a dataframe with the map.from and map.to columns, while the generic **commandsPOST** function relies on the composition of a command string using the idmapper parameters defined in Figure 2:

(RCy3 generic): commandsPOST(paste('idmapper map column,
                        columnName="name",  forceSingle="true", 
                        mapFrom="Ensembl",  mapTo="Entrez Gene", 
                        species="Human",  table="node",  sep=" '))

(RCy3 custom): mapTableColumn(column="name",  species="Human", 
                map.from="Ensembl",  map.to="Entrez  Gene")

A sample script demonstrates how to map identifiers via RCy3, covering the most common use cases ( https://github.com/cytoscape/RCy3/blob/master/vignettes/Identifier-mapping.Rmd).

### Case 1: Species-specific considerations

The Yeast Perturbation sample network provided with Cytoscape can be loaded from the Starter Panel and provides gene identifiers of the form “YDL194W”. These are actually Ensembl-supported identifiers for Yeast, distinct from the typical “ENSXXXG00000123456” form as presented in Table 1. This presents a special case that users will need to be aware of when selecting species and source database or *mapFrom* in the GUI. (Ensembl has special cases for Yeast, Worm and Fly identifiers in addtition to the standard terms that start with ENS.) In terms of automation, you could generate a new column of Entrez Gene IDs in this network with these calls:



(RCy3): mapTableColumn(column="name", species="Yeast", 
                map.from="Ensembl", map.to="Entrez Gene")

(py2cytoscape): cyclient.idmapper.map_column(source_column="name", species="Yeast", 
                source_selection="Ensembl", target_selection="Entrez Gene")

### Case 2: From proteins to genes

When working with protein interaction networks, for example those from the STRING database (see http://apps.cytoscape.org/apps/stringapp), you may want to translate protein identifiers (e.g., Uniprot-TrEMBL) to gene identifiers. The idmapper app supports this case as well, but one should be aware of the assumptions involved when making this translation. Since most genes encode for many proteins, you may have many-to-one mappings in your results. For all human networks imported from STRING using the *StringApp* ^5^, the following commands will perform an ID mapping from Uniprot-TrEMBL (proteins) to Ensembl (genes):

(RCy3): mapTableColumn(column="canonical name", species="Human",
                map.from="Uniprot–TrEMBL", map.to="Ensembl")
                
(py2cytoscape): cyclient.idmapper.map_column(source_column="canonical name",
                species="Human", source_selection="Uniprot–TrEMBL",
                target_selection="Ensembl")

### Case 3: Identifiers and symbols

In contrast to gene names and symbols, identifiers provide a more reliable means of specifying a particular gene. All data integration should be performed using identifiers as keys. Nevertheless, names and symbols play an important role in making results easier to read and understand. The idmapper app is primarily concerned with identifiers. However, relying on a subset of commonly used sources from BridgeDb ( Table 1) it does provide one exception. HGNC symbols, when used properly, can serve as identifiers in ID mapping and more generally can be added when starting from any other human ID source:

(RCy3): mapTableColumn(column="canonical name", species="Human", 
                map.from="Ensembl", map.to="HGNC")
(py2cytoscape): cyclient.idmapper.map_column(source_column="canonical name", 
                species="Human", source_selection="Ensembl",
                target_selection="HGNC")

## Limitations

The idmapper app provides easy access to a critical subset of ID mapping functionality originally covered by the BridgeDb app. When users run into the limitations of idmapper, they still have the option of installing and using the full-featured BridgeDb app from https://apps.cytoscape.org/apps/bridgedb. Examples of limitations include support for additional species or data sources. The BridgeDb app includes more of both as well as means to access custom data sources.

## Software availability

1. Software available from the Cytoscape App Store: http://apps.cytoscape.org/apps/idmapper
2. Latest source code: https://github.com/cytoscape/idmapper
3. Archived source code as at the time of publication: https://doi.org/10.5281/zenodo.1246814 ^6^
4. License: Apache License, Version 2.0: http://www.apache.org/licenses/LICENSE-2.0.html
