# Supplementary material for: Identifier Mapping in Cytoscape
Source: F1000Res. 2018 Aug 6;7:725. Originally published 2018 Jun 11. [Version 2] doi: 10.12688/f1000research.14807.2 (PMC6053696; doi:10.12688/f1000research.14807.2)
Supplement: Peer review history

# Peer review: Referee response for version 2

**DOI:** 10.5256/f1000research.16859.r36844

**Published:** 2018-08-08

**Authors:** Nadezhda T. Doncheva

**Affiliations:** 1 Novo Nordisk Foundation Center for Protein Research & Center for non-coding RNA in Technology and Health, University of Copenhagen, Copenhagen, Denmark

The idmapper is a simple, but very useful app for Cytoscape that significantly enhances the functionality of Cytoscape for users of the GUI and the CyREST interface. My concerns about the article have been addressed by the authors and I am very glad to hereby approve the revised version.

I have read this submission. I believe that I have an appropriate level of expertise to confirm that it is of an acceptable scientific standard.

# Peer review: Referee response for version 2

**DOI:** 10.5256/f1000research.16859.r36842

**Published:** 2018-08-07

**Authors:** Ruth Isserlin

**Affiliations:** 1 Donnelly Centre for Cellular and Biomolecular Research, Donnelly Centre for Cellular and Biomolecular Research, University of Toronto, Toronto, ON, Canada

This version addressed all my concerns.  The idmapper app will be a very user friendly, and useful addition to Cytoscape

I have read this submission. I believe that I have an appropriate level of expertise to confirm that it is of an acceptable scientific standard.

# Peer review: Referee response for version 1

**DOI:** 10.5256/f1000research.16116.r34907

**Published:** 2018-07-19

**Authors:** Nadezhda T. Doncheva

**Affiliations:** 1 Novo Nordisk Foundation Center for Protein Research & Center for non-coding RNA in Technology and Health, University of Copenhagen, Copenhagen, Denmark

The paper describes idmapper, a simple yet very useful app for converting node identifiers from one data source to another. It is provided as part of the widely used network analysis and visualization software Cytoscape and the mapping functionality can be used by users in several different ways, including the GUI, Cytoscape commands, and from R or Python scripts vie the Cytoscape REST interface. It supports common data sources such as UniProt and Ensembl. Both the app and the manuscript are in good quality and there are a few minor aspects to be addressed.

- In the Implementation section, it is important to clarify which extent the idmapper app relies on the BridgeDB mappings/app and also say a few words about how these mappings are done in BridgeDB and what the versions of the used data sources are. Maybe it can be a subsection called Dependencies or Backend?
- In the use Cases section, it would be useful to provide the Cytoscape command call in addition to the R and python function calls.
- Figure 2 is not very informative as it is now. Maybe it would be better if it includes a screenshot of the “Example Value” code? In addition, the term “singular function” is used in mathematics and might be confusing for some readers.

The app works nicely both through the Cytoscape GUI and the command/swagger interface. However, it would be great if the documentation can be improved a little bit so it is more consistent and informative. In particular:

- Could a list of all possible species and all possible data sources names be provided both in the manuscript and in the documentation of the command in Cytoscape/Swagger? It could be included as part of the description of the map column function in the Cytoscape automation section.
- The species are most likely the ones listed in the species parameter description but it is clearly stated anywhere. Is there a comma missing in the sentence: ”The combined common or latin name”? It seems that one can use all three of those: the common name, the latin name or the combined one (although there is a warning if anything but the combined one is used).
- Are the data sources exactly and only the ones in Table 1? There is an example with “Uniprot” (GUI, swagger documentation and example), one with “Uniprot-TrEMBL” (Case 2 in the paper) and in the table it is written “Uniprot TrEMBL”. Are all of those the same or not and if not what are the differences?
- For the table, it says “node” in the command documentation and “default node” in the swagger example. Both of them work, but it would be good to have a list of possible values as it is the case for the species parameter.
- Make clear (maybe in table 1) that Ensembl refers to Ensmbl Gene identifiers and not Proteins.
- Would it be possible that the parameters of the python function have the same names as the parameters in all other functions? Only recommended if it does not break already existing scripts.

There were two minor issues while testing the app:

- When running the following command from within Cytoscape or the swagger documentation on the galFiltered network (or on a STRING network), a warning and an error message come up in the Task Manager. It seems to work nonetheless, so could you check what is going on there?
- idmapper map column columnName="name" mapFrom="Ensembl" mapTo="Entrez" species="Yeast"
- warning: value not contained in list of possible values possible items = [] and error: networkTable not found.
- The data source inferring works well for the identifiers, but in the case that the species is wrong (e.g. switching from a yeast to a human network), changing the species resets the Map from column to the first entry in the list.

I have read this submission. I believe that I have an appropriate level of expertise to confirm that it is of an acceptable scientific standard, however I have significant reservations, as outlined above.

# Response

**Published:** 2018-07-22

**Authors:** Alexander Pico

**Affiliations:** The Gladstone Institutes, UCSF, USA

Nadya, thank you for the review. We have prepared a version 2 of the paper that addresses your main comments and corrections. A couple additional notes that are not reflected in the updated text:

- The R and Python library projects are independently developed, so coordinating on parameter names is impossible and in many cases not desired since consistency within each is much more important than across them, i.e., most users will pick one or the other and want maximum consistency with the conventions of that language, etc.
- Figure 2 is a requirement for all Cytoscape Automation papers. Even for cases like ours where there is just one operation.  It's our fault for making such a simple app!
- Thanks for the bug reports. We will try to reproduce and fix these for future releases of the app.

# Peer review: Referee response for version 1

**DOI:** 10.5256/f1000research.16116.r34906

**Published:** 2018-07-03

**Authors:** Augustin Luna

**Affiliations:** 1 cBio Center, Department of Biostatistics and Computational Biology, Dana-Farber Cancer Institute, Boston, MA, USA

This Cytoscape app provides functionality that is widely useful for Cytoscape users for converting network identifiers to different databases. Some points not completely clear:

1. The list of regular expressions used for inference, do they come from identifiers.org? What happens if the inference fails? Does the app try to pick the closest matching regular expression?
2.  For use of RCy3, is RCy3 a generic package to interact with any REST function in any Cytoscape package? Or did the developers of RCy3 specially include the mapTableColumn function to access the idmapper app? 

I have read this submission. I believe that I have an appropriate level of expertise to confirm that it is of an acceptable scientific standard.

# Response

**Published:** 2018-07-03

**Authors:** Alexander Pico

**Affiliations:** The Gladstone Institutes, UCSF, USA

Augustin, thanks for the review.

1. The regular expressions come from BridgeDb (which in turn gets them from identifiers.org). If there isn't a match to the regular expressions (or if more than one system is matched), then it just picks the first option in the list. A few of the system types aren't well specified. It's a simple matter to override this in the UI. We will add a sentence or two in the next version of the paper in response to all reviewers.

2. Right. RCy3 supports both a generic function call and a specific mapTableColumn function call. Since this is a "core" app, the RCy3 package supports custom convenience functions to make this operation easier to use and better documented.  We will add a more detailed description and contrasting example in the next version of the paper.

# Peer review: Referee response for version 1

**DOI:** 10.5256/f1000research.16116.r34908

**Published:** 2018-06-12

**Authors:** Ruth Isserlin

**Affiliations:** 1 Donnelly Centre for Cellular and Biomolecular Research, Donnelly Centre for Cellular and Biomolecular Research, University of Toronto, Toronto, ON, Canada

The paper entitled " **Identifier Mapping in Cytoscape: idmapper**" by Adam Treister and Alexander Pico presents a new implementation of id mapping available directly through Cytoscape with no additional configuration.  

 There is a lot of discussion throughout the paper about BridgeDB.  It is not clear what the relationship between idmapper and BridgeDB is.  It is understood that idmapper is a simplification and alternative of the aforementioned tool but does idmapper rely on BridgeDB?  Do they share a codebase?

 One thing that might be a good addition to the implementation section is a short discussion on the backend.  Where are the mappings coming from?  Is the app dependent on any online resources or are the mappings static and stored within the Cytoscape instance?  This is related to the previous BridgeDB question as given that there is no discussion about the backend I thought maybe the info was coming from BridgeDB and that was the reason it was included.

 In the implementation section, Table 1,

- it might be useful to have a column specifying the data source names that the app recognizes.  (for example in the table one of the data sources listed is "Uniprot TrEMBL" and later in Case 2 in the command the map.from(RCy3) and source_selection(py2cytoscpae) the db is specified as "Uniprot **-**TrEMBL" (with a dash)). 
- Given that this is the implementation section it might be good to list the regular expressions that each identifier recognizes (provided that they aren't too messy) and any exceptions they look for. 
- Later on in the case 1 you mention an exception to the basic regular expression behaviour for yeast.  Are there more exceptions that the app handles? 
- It is also unclear why the "Code" column is required.
- Might be nice to separate the data sources that can handle any species and those that are species specific.

In the use cases section "The default ***Species*** is determined by the previous selection made per network, providing a "smart and sticky" behavior. "  It is unclear but the previous selection was?

 In the use cases section in the specific cases two example use cases are presented, species specific and protein to gene conversions.  It would be helpful to list these and other common use cases at the start of the use case section as well.  One of the most common use cases being going from non-descriptive identifiers (like entrez gene ids, and ensembl ids) to something more understandable such as species specific IDs (HGNC or MGD,  Is it possible to map to proper gene symbols?)

 In the use case section it is stated that if the ID maps to multiple identifiers there is an option, " *Force single*", that when selected the app selects the first result.  How are the returned IDs sorted?  Is the first match the "best" match, alphabetical, random?

 In the Cytoscape automation section in the parameters section for the species option all the available mappings are listed but for the mapFrom and mapTo no options are listed.  (if the recognized data source name is add to Table 1 you can just reference the table here or if the first column of Table 1 are the recognized names it would be good to reference it here).  Also, the parameters listed for Cytoscape automation section are very different from the parameters used in the use cases which can be very confusing.  Maybe adding an example using the RCy3 commandsGet option under RCy3 and py2cytoscape examples just showing how the user can use all the parameters as specified using the command directly. 

**Minor comments/questions:**

- In the introduction "Uniprot and various specied-specific sources" should be "Uniprot and various specie **s**-specific sources"
- In the implementation section "The app looks at the first ten entries and choose the source" would be better as "The app looks at the first ten entries and choose **s** the source"
- In the implementation section - "This number of identifiers iteratively sampled is set by a static variable called *N_Iterations*. The algorithm for inferring the data source is implemented in *IdGuess.java*." - This is a little confusing why this is needed.  Is this a parameter the user can control or tweak?
- In Use cases section - "As such it usage in Cytoscape via" should be "As such it **s** usage in Cytoscape via"
- In use case 2 "you may want to translate to gene identifiers" might be better as "you may want to translate **protein identifiers (for example: Uniprot-TrEMBL)** to gene identifiers"
- Can idmapper convert a list column? (in the example use case where the network is an enrichment map and each node contains a set of genes as opposed to each node being a gene)
- What is the resulting column name?  What if a column with that name already exists? 

I have read this submission. I believe that I have an appropriate level of expertise to confirm that it is of an acceptable scientific standard, however I have significant reservations, as outlined above.

# Response

**Published:** 2018-06-26

**Authors:** Alexander Pico

**Affiliations:** The Gladstone Institutes, UCSF, USA

Ruth, thank you so much for your thorough review. These clarifications, fixes and additions have greatly improved the article.  Version 2 should be released soon, addressing all the issues you raised. We decided not to include the regular expressions in Table 1, however. They are messy and are what you'd expect from the example identifiers provided, which we feel do a better job of communicating what to expect from each data source.

 We hope you'll have a chance to look over version 2 and find that it meets your expectations.
